# Experimental study of dye removal from industrial wastewater by membrane technologies of reverse osmosis and nanofiltration

**DOI:** 10.1186/1735-2746-9-17

**Published:** 2012-12-05

**Authors:** Mohammad Fadhil Abid, Mumtaz Abdulahad Zablouk, Abeer Muhssen Abid-Alameer

**Affiliations:** 1 Department Chemical Engineering, University of Technology, Baghdad, Iraq; 2 Ministry of environment, Baghdad, Iraq

**Keywords:** Membrane separation, Synthetic dyes, Reverse osmosis, Nanofiltration, Wastewater reuse

## Abstract

Currently, biological method has been utilized in the treatment of wastewater -containing synthetic dyes used by textile industries in Iraq. The present work was devoted to study the operating feasibility using reverse osmosis (RO) and nanofiltration (NF) membrane systems as an alternative treatment method of wastewater discharged from Iraqi textile mills. Acid red, reactive black and reactive blue dyes were selected, based on the usage rate in Iraq. Effects of dye concentration, pH of solution, feed temperature, dissolved salts and operating pressure on permeate flux and dye rejection were studied. Results at operating conditions of dye concentration = 65 mg/L, feed temperature = 39°C and pressure = 8 bar showed the final dye removal with RO membrane as 97.2%, 99.58% and 99.9% for acid red, reactive black and reactive blue dyes, respectively. With NF membrane, the final dye removal were as 93.77%, 95.67%, and 97% for red, black and blue dyes, respectively. The presence of salt (particularly NaCl) in the dye solution resulted in a higher color removal with a permeate flux decline. It was confirmed that pH of solution had a positive impact on dye removal while feed temperature showed a different image. A comparison was made between the results of dye removal in biological and membrane methods. The results showed that membrane method had higher removal potential with lower effective cost. The present study indicates that the use of NF membrane in dye removal from the effluent of Iraqi textile mills is promising.

## Introduction

Large quantities of wastewater which contains toxic organic residues are generated from the textile and dye manufacturing processes. Synthetic dyes are considered the most difficult to treat because they contain complex aromatic molecular structures, which make them more stable and more difficult to be biodegraded [1,2]. Due to their chemical structure, dyes are resistant to fading on exposure to light, water, and many chemicals [3].

There are many structure varieties such as acidic, basic, disperse, azo, diazo, anthroquinone based, and metal complex dyes. These dyes are very stable and can be decomposed only at temperatures higher than 200°C. For this reason, synthetic dyes often receive considerable attention from researchers in textile wastewater treatment processes [4].

The present work will focus on separation by two types of pressure-driven membranes which are reverse osmosis (RO) and nanofiltration (NF) membranes. NF is characterized by a membrane pore size between 0.5 and 2 nm and operating pressures between 5 and 40 bars. It is used to achieve separation between sugars, other organic molecules and multivalent salts on one hand and monovalent salts, ions and water on the other. RO or hyperfiltration is characterized by a membrane pore size in the range of 0.5 nm. The operating pressures in RO are generally between 7 and 100 bars. The importance of these membrane processes can be judged from the membrane area installed in various industrial sectors. The ability of RO membranes to remove both organic and inorganic compounds has made it attractive for the treatment of contaminated drinking water supplies [5]. Reverse osmosis processes can simultaneously remove hardness, color, many kinds of bacteria and viruses, and organic contaminants such as agricultural chemicals and trihalomethane precursors.

[6] stated that a combination of NF/RO for nitrate removal would suffer less from scaling than a single RO because of CaSO_4_ and CaCO_3_ removal in the NF step. Cristiane *et al.*[7] studied the application of nanaofiltration process mainly in the rejection of color and chemical oxygen demand (COD) present in textile industry wastewater. The results of the tests showed the values for color rejection were around 99% and 87% for COD rejection. The process was efficient and promising for the reuse of wastewater for this type of industry. Al-Aseeri [8] investigated the removal of sodium chloride and acid red dye from aqueous solutions. Three acid dye concentrations (0.10, 100 and 200 mg/L) and three NaCl concentrations (100, 1000 and 5000 mg/L) were used. Results showed that in the absence of NaCl, color removal of 97.2% was achieved and this number was elevated to 98.2% at dye concentration of 200 mg/L when 1000 mg/L NaCl was added to the colored water.

[9] investigated the effluents from the cotton textile industry which were treated by nanaofiltration membrane in order to reduce the quantity of the disposed water and at the same time to reuse the treated water. Results showed that NF membranes could achieve complete decolorization of the cotton dye effluent and reduced the total salt concentration more than 72%. These membranes can be used even at high recoveries and reasonably low pressures, producing high quality water, which can be reused. [10] investigated the treatment of secondary effluent for wastewater reuse in the textile industry; in their work, they used (NF90) membrane. Results showed that NF90 yielded a COD reduction of 99% and the highest salt rejection (75%-95%). As the permeate quality was obtained, the levels of COD removal and salt rejection were not significantly affected by fouling and that high flux percentage could be retrieved after cleaning.

The rejection efficiency of organic and salt decreased with the decrease in pH, because of osmotic pressure increase, leading to permeate flux decline and decrease in salt passage. In addition, the improved salt rejection was likely due to Donnan exclusion by humic material close to membrane surfaces. The average rejection efficiency of humic acid and salt ranged between 91.2%-95.25% and 63.6%-80%, respectively.

Currently, biological method has been utilized in the treatment of wastewater containing synthetic dyes used by textile industries in Iraqi. The present work is devoted to study the operating feasibility of pressure-driven membrane system as an alternative treatment method of such a wastewater.

## Materials and methods

### Experimental apparatus

**Figure 1:**
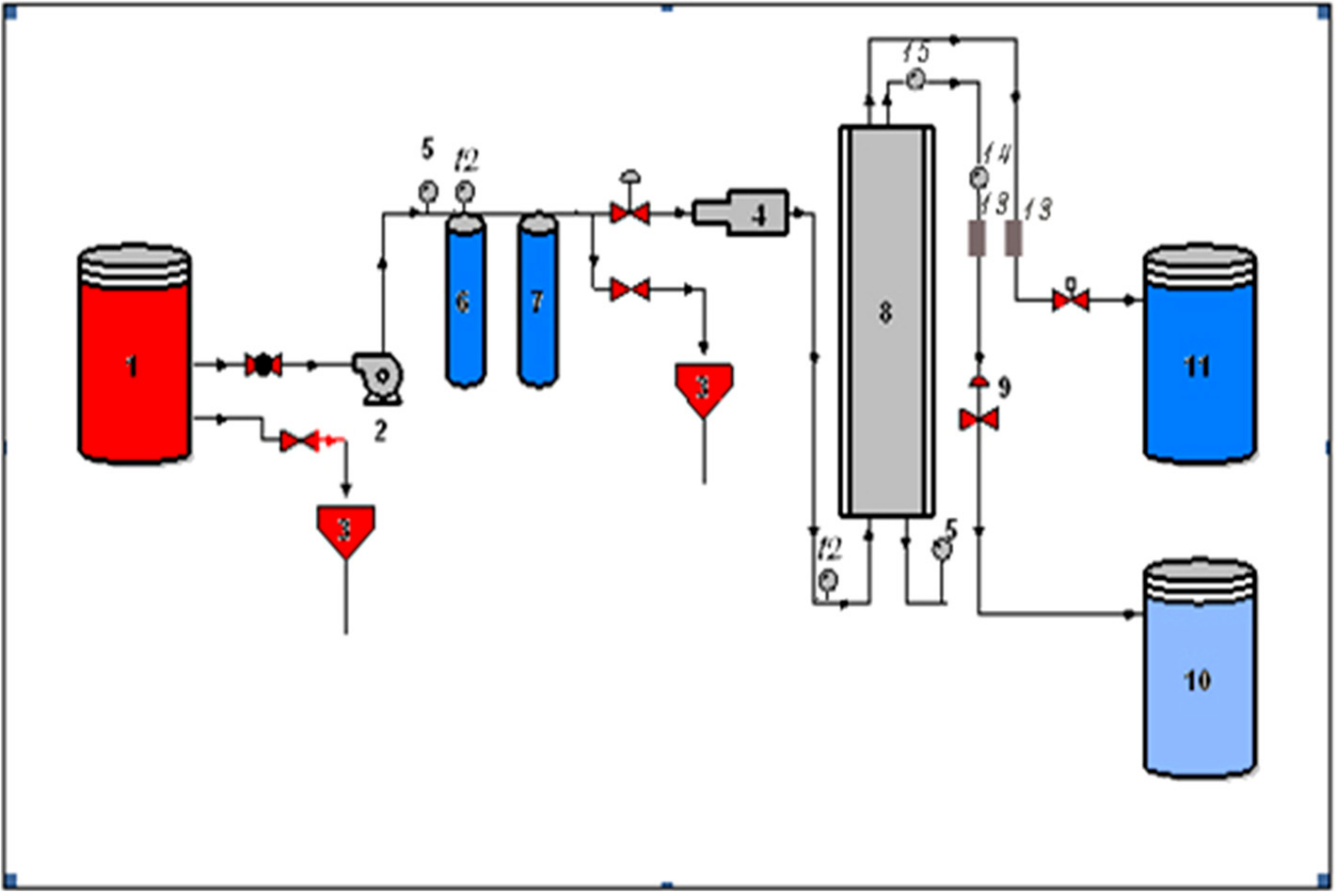
**Schematic diagram of the pilot plant. **1. Feed water tank, 2. Feed water pump, 3. Drain, 4. High pressure pump, 5. Pressure gauges,6,7-Microfilter (5 μ,1 μ), 8. Pressure vessel for RO or NF Membrane, 9-Regulating valve, 10-Permeate Tank, 11. ConcentrateTank, 12-Temperature Gauges, 13-Rotameters, 14-Condictivity meter, 15-TDS meter.

All experiments were conducted on a pilot plant scale. A test skid unit was arranged, shown schematically in Figure 1.

Two types of membranes, (RO) and (NF) were used. Each membrane was mounted in turn into stainless steel housing. The characteristics of RO and NF membranes are shown in Table 1. The liquid was circulated through the pilot plant by two pumps, the low pressure pump (Type: centrifugal, Model: CEAM, Q = (30-100 L/min), H = 20-30 m) which delivered the liquid solution from the feed tank to the suction of the high pressure pump (Type: multistage, Model: p1TW10A02) via microfilters of 5 μm and 1 μm, respectively. The function of the microfilters was to get rid off suspended solids and decreasing turbidity and silt density index (SDI) in the influent line to the membrane chamber.

**Table 1:** **The Characteristics of RO and NF membranes (**http://www.membranes.com**)**

**Type of membrane**	**RO**	**NF**
Model	ESPA1-4040	ESNA1-LF2-4040
Material	Composite polyamide	Composite polyamide
Module	Spiral wound	Spiral wound
Size(I.D*Length)(inch)	Inch(4 × 40)	inch (4 × 40)
Active Area,m^2^	7.9	7.9
Max operating temp,°C	45	45
Max Applied press, bar	41.4	21
Manufacture	Hydranautics	Hydranautics
Feed water pH Range	2-10	3-10
Maximum feed water Turbidity (NTU)	1.0	1.0
Maximum feed water SDI (15 min)	5.0	5.0

The pressure across the membrane housing was supplied by the high pressure pump. The pilot plant contained three holding tanks, all were made of polyethylene. These were the feed tank (500 L), the concentrate tank (500 L) and the permeate tank (125 L). Each tank was supplied with suitable fitting and connections to serve the process.

Flow rate, pressure and temperature of the flowing streams were measured as follows. Two calibrated rotameters were used to measure the flow rate of the permeate and concentrate. The pressure was measured by liquid filled bourden type gauges. Temperatures at upstream and downstream of the membrane housing were measured by dial gauge type temperature indicators with sensors type (Pt/100). The pilot plant was also supplied with two on-line instruments attached to the unit to measure electrical conductivity (EC) and total dissolved solids (TDS) of permeate and concentrate, respectively. In addition to the skid mounted conductivity and TDS meters, laboratory portable conductivity TDS and pH meters from Hanna-USA were used for further check and quick measurements.

### Chemicals

Three types of dyes that are mostly used in Iraq were selected for the treatment investigation. These dyes were supplied from 14-Ramadhan factory of textile industries, Ministry of Industry, Baghdad, Iraq. The characteristics of these dyes are presented in Table 2. Analytical grade chemicals HCl 36%, H_2_SO_4_98% and NaOH were used to adjust feed pH and to clean the membranes.

**Table 2:** Characteristics of applied dyes used in the experiments (14-Ramadhan textile mill)

**Type of dye**	**Commercial Name**	**Mwt gm/gmol**	**Chemical structure**	***λmax *(nm)**
Acid red	Lancron	696.66	C_32_H_22_N_6_Na_2_O_6_S_2_	500
Reactive black	Forosyn Black (SER)	991.82	C_26_H _21_N_5_Na_4_O_19_ S_6_	600
Reactive blue	Forosyn Blue (SE)	1309.87	C_38_H_28_C_12_N_14_O_18_S_5_Na	570

### Experimental procedure

Experiments were carried out in different steps: In the first step, each dye solution was prepared in four concentrations of 50, 55, 60 and 65 mg/L in order to study the effect of dye concentration on rejection coefficient of the membrane. The preparation was done in the feed tank by mixing of every individual dye powder with distilled water. In the second step, the experiments were carried out at different pressures (6, 8, 10 and 12 bars) for each individual concentration of every dye. In the third step, four different feeds pH (4.5, 6.0, 7 and 8.3) were investigated. The feed pH was adjusted by addition of HCl and NaOH to the feed tank. In the fourth step, two different temperatures were tested (39°C and 26°C) to investigate the seasonal effect on the membrane performance. Steps 1, 2, 3 and 4 were repeated using tap water as solvent to study the effect of TDS on dye removal. Sample analysis of wastewater effulent from textile mill is shown in Table 3. Table 4 shows the physicochemical analysis for tap water used in the membrane testing system. In all steps, samples were collected for analysis every 15 minutes. All experiments were carried out in 2h to reach the steady state conditions.

**Table 3:** Analysis of wastewater effluent from the textile mill

**Parameter**	**Units**	**Measured values during the tests (2010)**
pH		5 – 8
TDS	mg/L	400-1000
Conductivity	μs/cm	700-1300
Range of dye concentration^*^	mg/L	20 - 50

*- considering each type of dye separately.

**Table 4:** Physicochemical analysis for tape water used in membrane testing system

**Parameter**	**Units**	**Iraqi Drinking water guideline (2010)**	**Measured values during the tests (2010)**
Turbidity	NTU	5	5
pH		6.5-8.5	7.4 – 8.0
TDS	mg/L	1000	800 -2000
Elec. Cond.	μS/cm		1400 - 3700
T. Hardness	mg/L as CaCO_3_	500	590 - 1500
Sodium (Na)	mg/L	300	90 - 240
Potassium (K)	mg/L		2.6 – 4.0
Calcium (Ca)	mg/L	50	88 - 220
Magnesium (Mg)	mg/L	50	57 - 160
Chloride (Cl)	mg/L	250	270 - 900
Sulphate (SO_4_)	mg/L	250	90 - 240
Bicarbonate	mg/L		177- 230
Turbidity	NTU	1.4	1-2
SDI		0.5	0.4-0.6

### Analytical methods

**Figure 2:**
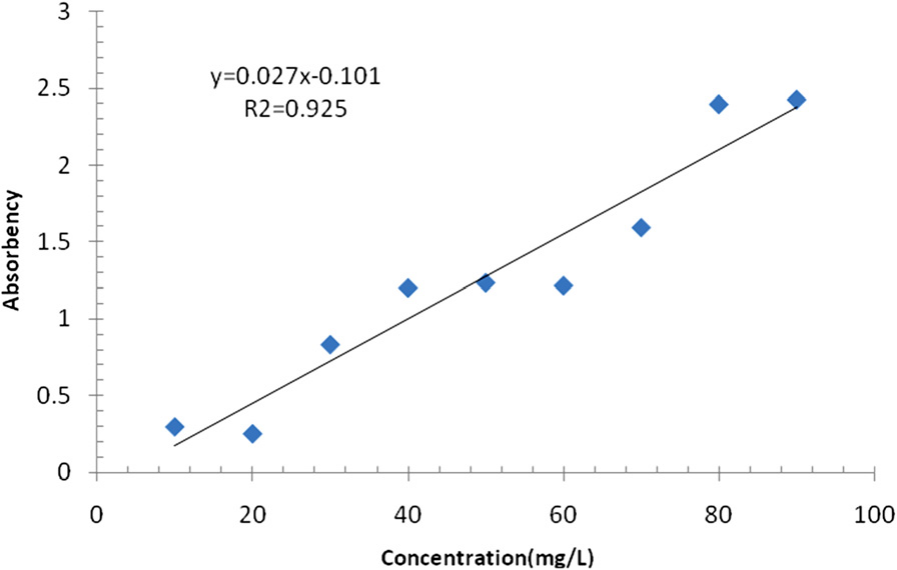
Calibration curve of Acid red dye using UV-spectrophotometer.

Analysis of samples was carried out based on standard methods. The color which is a function of dye concentration was determined spectrophotometrically at a dominate wavelength by spectrophotometrically method No.2120 of Standard Methods, using a Shimadzu UV-visible spectrophotometer (UB-1201 PC) which measures the light absorbency of a dye solution. Figure 2 illustrates the calibration plot of light absorbency vs. different dye concentrations of the prepared solutions. The solution conductivity was measured by portable conductivity meter (Horiba DF-H). Samples were measured by portable pH meter (HACH digital pH meter). Retention factor (R) of each species was calculated [11] as:

(1) $$\%R=\left(1-\frac{Cp}{CR}\right)\times 100$$

Where R is rejection factor (%), C_p_ is the solute concentration in the permeate (mg/L), C_R_ is the solute concentration in the feed solution (mg/L), and permeate flux (J_w_) of the membrane is calculated as:

(2) $$J_{w}=\frac{Q_{p}}{A}$$

Where the J_W_ is the permeate flux (L/m^2^.h), Q_P_ is the permeate flow rate per hour and A is active surface area of membrane (m^2^).

## Results

**Figure 3:**
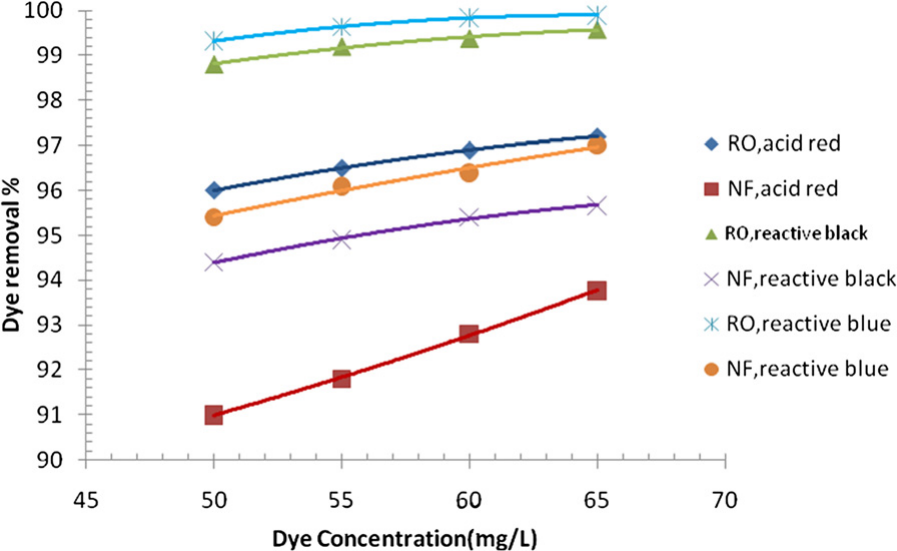
Effect of dye concentration on dye removal of RO and NF membrane, (p = 8 bars, pH = 8.3, T = 39°C).

**Figure 4:**
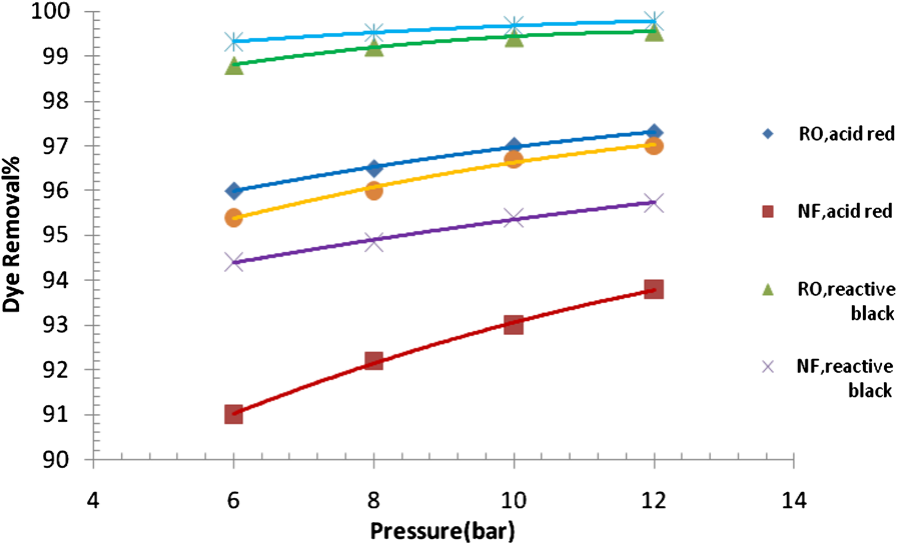
Effect of membrane pressure on dye removal of RO and NF Membrane (pH = 8.3 T = 39°C).

**Figure 5:**
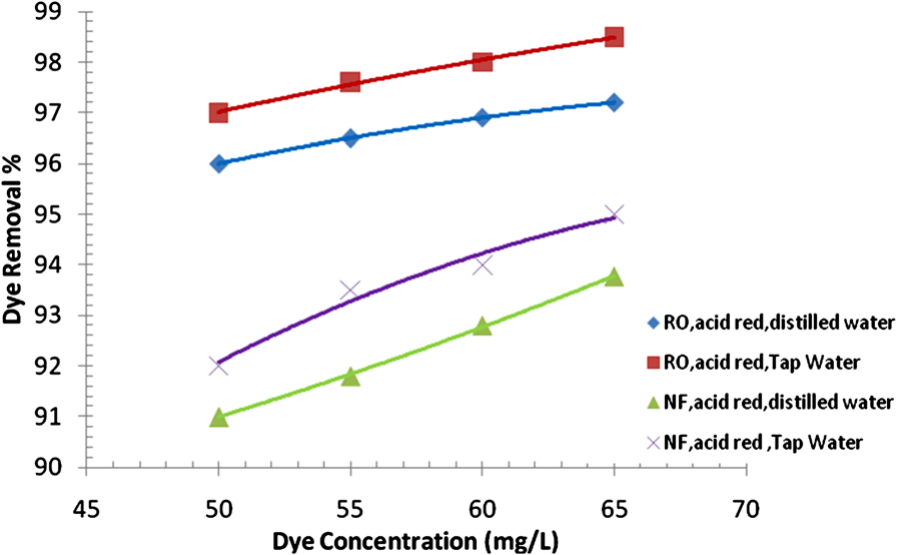
**Effect of dye concentration on acid red dye removal of RO and NF membrane with tap and distilled water.** (p = 10 bars, pH = 8.3, T = 39°C).

**Figure 6:**
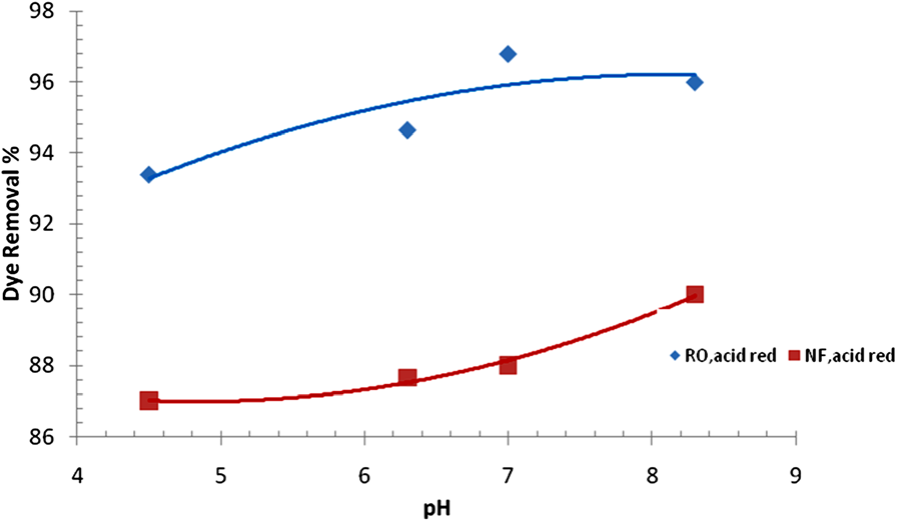
Effect of pH on acid red dye removal of with RO and NF membranes (C = 50 mg/L, p = 8bars and T = 39° C).

**Figure 7:**
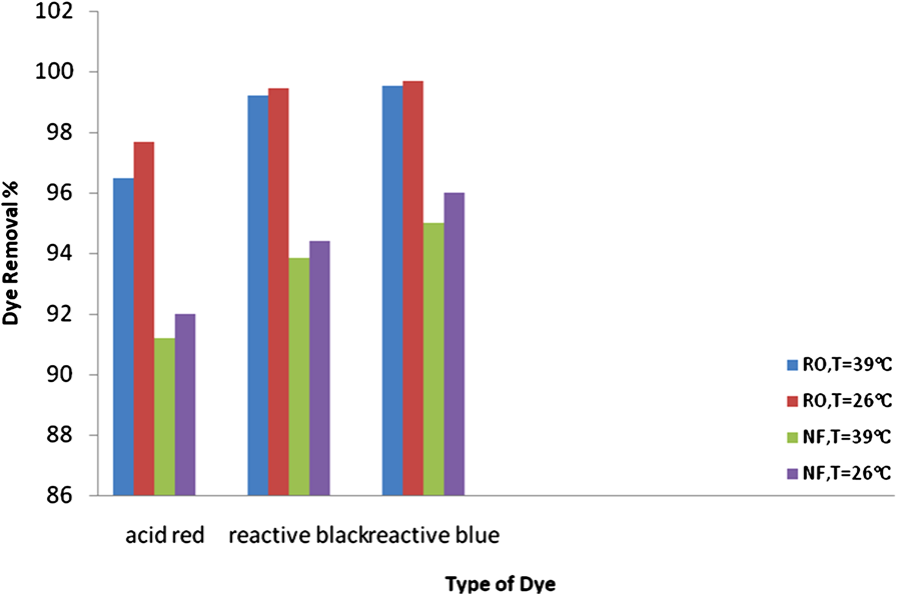
Effect of feed temperature on dye removal with RO and NF membranes at different types of dye (p = 8 bar, pH = 8.3 and C = 50 mg/L).

**Figure 8:**
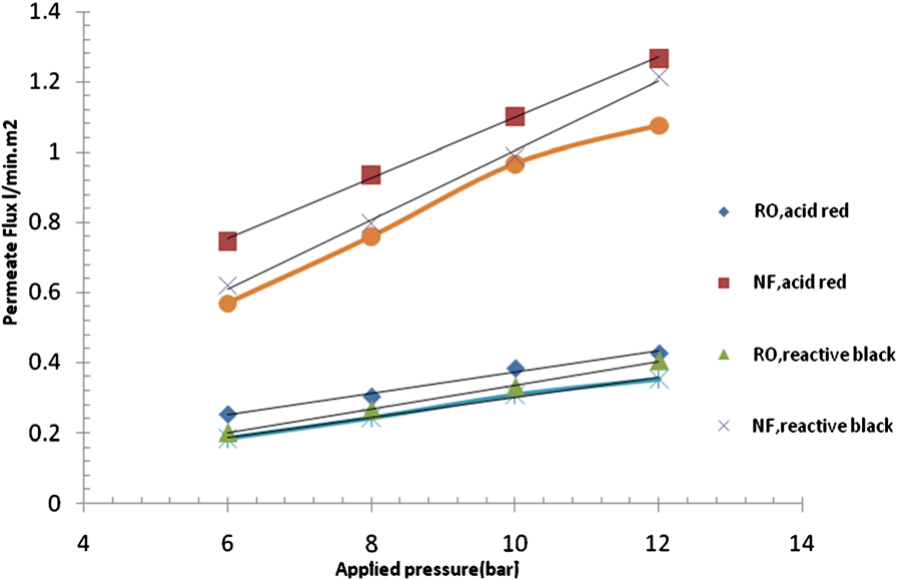
Effect of applied pressure on permeate flux of RO and NF membranes using different types of dye (C = 50 mg/L, pH = 8.3 and T = 39°C).

**Figure 9:**
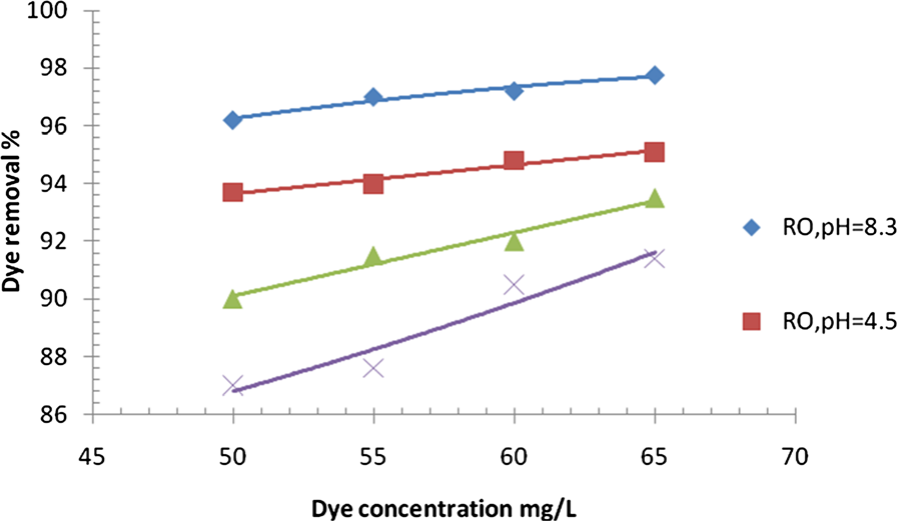
Effect of initial acid dye concentration on dye removal with RO and NF membranes at different pH of feed solution (p = 8 bars, T = 39°C).

Three types of dyes (acid red, reactive black and reactive blue) were selected, based on usage rate in Iraq. The effects of dye concentration, pH of solution, feed temperature, dissolved salts, and operating pressure on dye removal and permeate flux were examined (Figures 3, 4, 5, 6, 7, and 8). Figure 9 presents a comparison for acid red dye removal between membrane separetion and biological method. Results and corresponding figures are presented in a way to view, on the same plot, the performance of RO and NF membranes utilized in the present work.

## Discussion

### Effect of dye concentration on dye removal

Figure 3 illustrates the variation of dye removal (R%) with dye concentration in feed for different type of dyes. As can be seen, the dye removal is positively related to the dye concentration. Results at operating conditions of pH = 8.3, feed temperature = 39°C and pressure = 8 bar showed that when dye concentration was increased from 50 mg/L to 65 mg/L, the dye removal with RO membrane was increased from 96% to 97.2% for acid red dye, respectively, and with NF membrane, the acid dye removal was increased from 93.77% to 97.2%, respectively. As expected, for higher feed concentration, higher dye removal was achieved. This is mainly due to the concentration polarization layer which is built on the membrane surface as a result of increasing dye concentration in feed and leading to higher osmotic pressure [12,13]. The system with RO membrane, in which the rejection can be affected more by size exclusion than other mechanisms, performs higher rejections than that for the system with NF membrane, as the effective hydrodynamic radius of dye molecule is typically larger than the membrane pore radius, the rejection of dye is therefore mainly controlled by sieving mechanism. Thus, it is less possible for dye molecules from passage through the membranes which have relatively smaller pore size, and NF has higher MWCO which means larger pore sizes than RO [14].

The higher dye removals were obtained for reactive black and blue dyes; this may be attributed to the low solubility characteristics of these dyes compared to that of acid dye. This lower solubility resulted in membrane fouling in the end of operation. Membrane fouling may be caused by the dye adsorption on the membrane surface observed at the experimental runs, which was indicated by the presence of color on the membrane after filtration [15].

### Effect of pressure on dye removal

Variation of dye removal against operating pressure is shown in Figure 4. As can be seen, dye removal followed a positive trend with the range of operating pressure studied in the present work. When operating pressure was increased from 6 bars to 12 bars, dye removal with RO membrane was increased from 96% to 97.3 for acid dye, respectively, and with NF membrane, dye removal was increased from 91% to 93.7% for acid dye, respectively. This may be attributed to mechanical compaction of membrane at higher operating pressure. Compaction is the decrease in membrane volume due to mechanical deformation upon the application of a high mechanical pressure. A change in density of the active layer of the membrane implies a change in free volume available. This will have a significant influence on transport of permeating components [16].

Mechanical compaction will normally yield an increase in the density of membrane material and decrease in pore size which will decrease the rate of diffusion of dissolved solute leading to an increase of dye removal. Many researchers of the field [17,18] have studied this reversible phenomenon. Their findings were in agreements with our results.

### Effect of salt concentration on dye removal

NaCl is one of the most common inorganic salts that have been widely used in dyeing process for the purpose of enhancing the degree of dye fixation onto fabric. The dissolved salt in waste stream must be treated properly before being discharged into environment [14]. Figure 5 illustrates the performance of RO and NF membranes for dye removal against acid red dye concentration, using distilled water and tap water, respectively. All the experiments were carried out for 2 h, to reach the steady state conditions. As expected, increasing the salt concentration resulted in higher dye removal. This may be due to the osmotic pressure of solution which increases with salt concentration and consequently a concentration polarization layer will be built up by the salt that acts as an additional barrier to the passage of the color, it seems that the effect of concentration polarization was more for membrane with smaller pore size (i.e., RO membrane) in which case of fouling can be significant [19].

Another contribution concerns the electrostatic behavior of NF membrane which has a surface of slightly negative charge due to the sulfonic acid groups R-COO^-^ (which is responsible for rejection of Cl^-^ ions in dillute electrolyte solution). This electrostatic repulsion made the negative ions accumulate near the membrane surface accelerating the formation rate of the concentration polarization layer. Thus the separation performance of NF membrane system was found to be significantly dependent on the steric and charge effects. This right combination of membrane pore size (steric effect) and its effective charge density (Donnan effect) may lead to an optimum separation performance [20].

### Effect of feed pH on dye removal

It has been acknowledged that alkaline environment always has the best condition for enhancing the degree of dye fixation during dying process, though acidic condition would also be considered for certain textile operation [14]. Figure 6 demonstrates the variation of acid red dye removal against pH of solution with RO and NF membranes, respectively. The plot illustrates a positive trend between the two variables with both membranes. It is well known that when pH of a solution decreased, the solubility of present salts increased. When pH of solution was increased from 4.5 to 8.3 pH, the dye removal with RO membrane was increased from 93.7% to 96.2% for acid dye, respectively, and with NF membrane, dye removal was increased from 87% to 90% for acid dye, respectively. From membrane point of view, decreasing pH of solution by addition of HCl acid would increase the solubility of salts and consequently decreases the rate of salt scaling on the membrane surface which leads to decrease the osmotic pressure of solution and consequently the dye removal decreases. On the contrary, increasing pH by addition of NaOH would accelarate the deposition rate of salt on the surface of the membrane. As mentioned ealier, surface of NF membrane has a slight negative charge, this will result an electrostatic repulsion force with OH^-^ ions for high pH solution. In higher pH, the electrostatic repellent force becomes strong and rejection will increase [21].

### Effect of feed temperature on dye removal

Feed temperature is another factor which affects the performance of (RO/NF) membranes. Figure 7 depicts the maximum dye removal resulted from increasing feed temperature with different types of dye, It shows that the increase in feed solution temperature results in lower dye removal. When feed temperature increased from 26°C to 39°C, the dye removal with RO membrane was decreased from 97.3% to 96.2% for acid dye, respectively, and with NF membrane the dye removal was decreased from 92% to 91% for acid dye, respectively. This may be attributed to the increase in the diffusion rate of the molecules across the external boundary layer and in the internal pores, owing to the decrease in viscosity of the dye solution in addition to increased membrane pore size [22]. This increase in pore sizes is partially characterized by higher dye passage. From Figure 7 it can be seen that the flux of acid dye is higher than that of reactive dye. This is due to the fact that molecules with smaller molar mass diffuse more easily than that of larger molar mass at the same operating conditions [23], and also to the fact that water permeability of the membrane increases with increasing temperature. These results are in agreement with the findings of [24].

### Effect of dye type and operating pressure on permeate flux

Figure 8 shows a comparison of the permeate flux between NF and RO membranes for variable feed pressure at different types of dye. It can be seen that higher flux values were obtained at 12 bars for any applied dye, since the increase in feed pressure will increase the driving force, overcoming membrane resistance [25]. On the other hand, the higher is the feed concentration, the lower is the permeate flux; this may be attributed to increasing the concentration polarization on the membrane surface and consequently increasing the osmotic pressure. When operating pressure was increased from 6 bars to 12 bars, permeate flux with NF membrane was increased from 0.76 to 1.25 (L/m^2^.h.) for acid dye, respectively, and with RO the flux was increased from 0.26 to 0.44 (L/m^2^.h.) for acid dye, respectively. Also it can be seen that the flux for the system with NF membrane is more than double that for the system with RO membrane. This is because NF membrane has larger pore size than that of the RO membrane.

The results shown in Figure 8 indicate that permeate flux of the acid dyes obtained were higher than that of the reactive dye at the same operating conditions. This is due to the decrease in solubility with the increase in molecular weight of dye applied. It should be noted that the mass increase may also be related to diffusion, because a bigger molecule will diffuse more slowly than a smaller molecule. The result of the present work seems to be in a good agreement with those observed by [26] for the effect of pressure at different dye concentrations.

### Effect of dye concentration and pH of solution on dye removal

The variation of dye removal (R%) with dye concentration in feed for different values of pH was illustrated in Figure 9. As expected when increasing both the concentration of the dye and pH in feed solution resulted in enhancement of dye removal. Results at operating conditions of pH = 4.5, feed temperature = 39°C and pressure = 8 bar showed that when acid dye concentration was increased from 50 mg/L to 65 mg/L, the dye removal with RO membrane was increased from 93.7% to 95.2% for acid red dye, respectively, and with NF membrane, the acid dye removal was increased from 87% to 91.5%, respectively. At pH = 8.3, the dye removal with RO membrane was increased from 96.2% to 97.7% for acid red dye, respectively, and with NF membrane, the acid dye removal was increased from 90% to 93.5%. Normally, a greater concentration of solutes leads to a greater polarization by concentration which, during the filtration, may lead to a higher degree of membrane clogging, which results in a greater rejection of solutes [27], while decreasing the pH from 8.3 to 4.5 increased the salt solubility in the feed of all the membranes (i.e., RO\NF), and resulted in decreasing the rate of formation of the polarization layer on the membrane surface, leading in lower osmotic pressure and consequently higher rate of mass diffusion through the membrane. Moreover, with NF the variation in pH may affect the permeability of the membrane by altering the interactions between the polymer chains of which it is composed. These alterations may increase or decrease the distance between the chains leading to an increase or decrease in the permeability of the membrane [28].

### Comparison between biological and membrane methods

**Figure 10:**
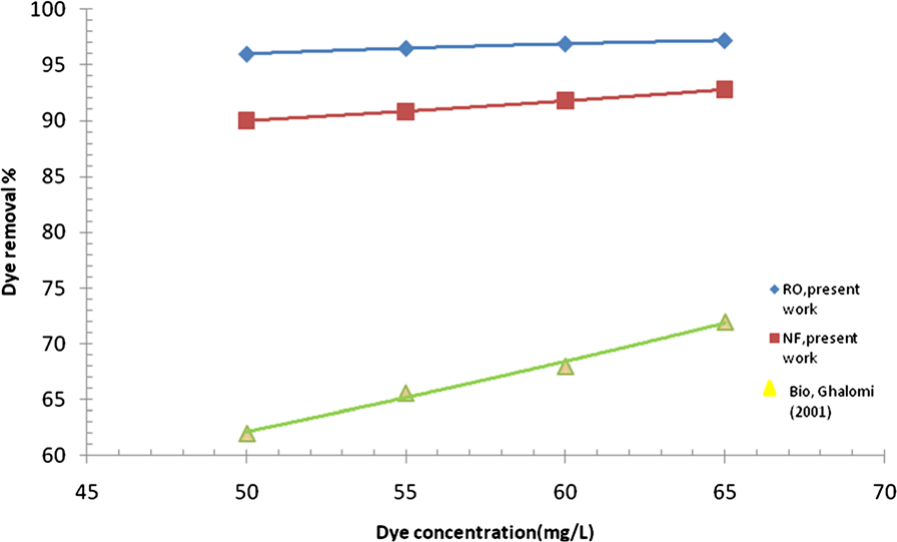
Effect of dye concentration on dye removal with (RO/ NF) membranes at (P = 8 bars, pH = 8.3, T = 39°C), and biological method.

A comparison was made between current biological treatment processes through ISO9888 method [29] and membrane separation system. The comparison criteria were between (R%) of water initially and after washing process, with (R%) of dye solution passing through the membrane in different dye concentrations. Figure 10 plots the removal potential of acid red synthetic dye as a function of dye concentration utilizing two methods of wastewater treatment. As can be seen, at operating conditions of p = 10 bars, pH = 8.3, and T = 39°C, the membrane method has the higher dye rejection with 98.5% for RO and 93.3% for NF. Biotreatment method offered maximum rejection performance of 74.6%. Hence, it seems that membrane separation system could be promising for dye removal from wastewater of textile industries in Iraq.

### The empirical correlations

The objective response value at each average seasonal temperature is the result of the interaction of several parameters, namely: operating pressure; dye concentration; pH; and TDS.

A power law formula was used to correlate the present experimental results of dye removal of acid red dye. Equation 3 is the proposed empirical correlation:

(3) $$F=a_{0}P^{a_{1}}C^{a_{2}}{\left(pH\right)}^{a_{3}}{\left(TDS\right)}^{a4}$$

Where: a_1_, a_2_, a_3_ and a_4_ are constants, representing the magnitudes of the effect of applied pressure, dye concentration, pH solution, and TDS, respectively, on the objective function (i.e., dye rejection), while a_0_ is a constant that depends on the nature of the operating system and the objective function. The constants of this correlation with the variance and correlation coefficient are shown in Tables 5 and 6 for acid red dye removed by NF and RO membranes, respectively. Tables 5 and 6 depict the order of effect of the operating variables on dye removal of NF and RO membranes in the following sequence: C > pH > P > TDS.

**Table 5:** Statistical analysis of fitting the experimental data for RO system (T = 39°C)

**Type of dye**	**Objective response**	**a**_**o**_	**a**_**1**_	**a**_**2**_	**a**_**3**_	**a**_**4**_	**Correlation factor (R)**	**Variance (V)**
Acid red	Dye removal	67.6889	0.0316	0.0563	0.0336	−0.0002	0.957	0.916

**Table 6:** Statistical analysis of fitting the experimental data for NF system (T = 39°C)

**Type of dye**	**Objective response**	**a**_**o**_	**a**_**1**_	**a**_**2**_	**a**_**3**_	**a**_**4**_	**Correlation factor (R)**	**Variance (V)**
Acid red	Dye Removal	38.3862	0.0466	0.1823	0.0496	−0.0089	0.984	0.969

## Conclusion

From the present work the following conclusions can be drawn: RO and NF membranes used in present study proved to be efficient tools to remove dye substance from effluent wastewater of Iraqi textile mills. Dye removal from wastewater was positively related to applied pressure, pH, TDS and dye concentration in feed solution, but it was inversely related to feed temperature. Applied pressure and solution temperature have positive impact on permeate flux from RO and NF membranes. But it was inversely related to dye concentration and pH. Wastewater with acid dye treated by RO or NF membranes result in lower rejection and higher permeates flux than wastewater with reactive dyes. It was found that the order of effect of the operating variables on dye removal of NF and RO membranes was in the following sequence: C > pH > P > TDS.

At the same operating conditions, one could get from NF system twice the permeated environmental accepted water flow rate and about 50% less electric power instead of RO membranes. The reduction of electric power came directly from the reduction of the operating pressure of the unit with NF membrane. Results indicated that the use of NF membrane in dye removal from wastewater of the Iraqi textile mills is promising and can be used with higher efficiency instead of the current biological method.

## Competing interests

The authors declare that they have no competing interests.

## Authors’ contribution

The authors’ qualifications, current positions they hold at the institution: MFA - qualification: PhD in chemical engineering-reaction kinetics and membrane separation. position: lecturer and researcher at the Chemical Engineering Department. MAZ - qualification: PhD in chemical engineering-mass transfer by adsorption. position: lecturer and researcher at the Chemical Engineering Department. AMA - qualification: MSc in chemical engineering- membrane separation. position: researcher in the Ministry of environment. All authors read and approved the final manuscript.
